# Penetrating Wounds of the Abdomen

**Published:** 1890-06

**Authors:** 


### PENETRATING WOUNDS OF THE ABDOMEN.

The question of operation in penetrating wounds of the abdomen is one that can no longer be lightly passed over by surgeons. The excellent articles by Prof. Senn, of St. Louis, which have appeared within the last two or three years have given a new impetus to this field of surgery, and now American surgeons, especially, are almost unanimous in favor of operating immediately instead of waiting until peritonitis has set in as has been the custom heretofore. The diagnosis of perforation of the intestines is invariably difficult, often impossible. Senn's method of inflating the intestines with hydrogen gas has not given as good results as were at first hoped for, and is rarely resorted to. An accurate diagnosis can only be made by an exploratory laparotomy—with our improved surgical methods a comparatively safe procedure—to which recourse should be had immediately when there is evidence of the abdominal cavity having been penetrated. Not infrequently the shock is slight, even where the intestines, or other viscera, have sustained serious injury. The patient should always be given the benefit of the doubt. The very improbable occurrence of recovery without operation—about one chance in a hundred—should not stay the hand of the surgeon. Nor is extreme shock, which is generally prolonged and increased by internal hemorrhage, a contraindication to an operation.

In by far the larger proportion of fatal cases death is the consequence of either hemorrhage, or septic peritonitis due to the escape of the intestinal contents into the peritoneal cavity. Hemorrhage can only be safely dealt with by ligating the bleeding vessels, and the surgeon or physician who expects to control dangerous internal hemorrhage by the administration of opium or the application of ice is certainly leaning on a broken reed. Septic peritonitis can be prevented only by opening the abdomen, suturing rents in the viscera and thoroughly flushing the peritoneal cavity.

With the decalcified bone plates of Senn, the segmented rubber rings of Brokaw or the cat-gut mats of Davis, resection of the intestines and intestinal anastomotic operations are very much simplified, and the length of time required for their performance decidedly shortened.

While the results thus far obtained are not brilliant, still they are encouraging, and lead us to hope for much better success, when the technique of the operations is more thoroughly mastered and the operations more promptly done.

